# Ethylene Vinyl Alcohol Copolymer Nanofibrous Cation Exchange Chromatographic Membranes with a Gradient Porous Structure for Lysozyme Separation

**DOI:** 10.3390/polym16081112

**Published:** 2024-04-16

**Authors:** Tianzhi Tang, Jinping Gan, Zhanrui Cao, Pan Cheng, Qin Cheng, Tao Mei, Liping Zhu, Feng Zhou, Ke Liu, Dong Wang

**Affiliations:** 1 Key Laboratory of Textile Fiber and Products, Ministry of Education, Wuhan Textile University, Wuhan 430200, China; tangtzhi@163.com (T.T.); wangdon08@126.com (D.W.); 2 Wuhan We-Change Technology Co. Ltd., Wuhan 430106, China; meitao006@foxmail.com; 3 State Key Laboratory for Modification of Chemical Fibers and Polymer Materials, College of Materials Science and Engineering, Donghua University, Shanghai 201620, China; zhulp@dhu.edu.cn; 4 Budweiser Brewing Company APAC, Wuhan 430051, China; feng.zhou@budweiserapac.com

**Keywords:** benzophenone, 2-Acrylamido-2′-methylpropanesulfonic acid, nanofiber membrane, membrane chromatography

## Abstract

Lysozyme, a common antimicrobial agent, is widely used in the food, biopharmaceutical, chemical, and medicine fields. Rapid and effective isolation of lysozymes is an everlasting topic. In this work, ethylene vinyl alcohol (EVOH) copolymer nanofibrous membranes with a gradient porous structure used for lysozyme adsorption were prepared through layer-by-layer nanofiber wet-laying and a cost-efficient ultraviolet (UV)-assisted graft-modification method, where benzophenone was used as an initiator and 2-acrylamide-2-methylpropanesulfonic acid as a modifying monomer. As indicated in the Fourier Transform Infrared (FTIR) and X-ray photoelectric energy spectrometer (XPS) investigation, sulfonic acid groups were introduced on the surface of the modified nanofibrous membrane, which possessed the ability to adsorb lysozyme. Compared with membranes with homogenous porous structures, membranes with a gradient porous structure present higher static (335 mg/g) and dynamic adsorption capacities (216.3 mg/g). Meanwhile, the adsorption capacity remained high after five cycles of the adsorption–desorption process. The results can be attributed to the gradient porous structure rather than the highest porosity and specific surface area. This suggests that the membrane with comprehensive separation performance can be designed from the view of the transmembrane porous structure, which is of significance for the development of next-generation advanced chromatographic membranes.

## 1. Introduction

Lysozyme is a common antimicrobial agent found in a variety of animals, plants, microorganisms, and higher vertebrates [1,2]; it is an enzyme that breaks down the 1,4-β bonds in the peptidoglycan layer of cell walls [3] and is widely used in food, biopharmaceuticals, and chemicals [4,5,6]. Egg white is the main raw material for lysozyme extraction. However, cleaning egg whites of impurities is a difficult task as a result of their low lysozyme levels. Therefore, rapid and low-cost access to lysozyme has long been a topic of inquiry [7].

The Russian botanist Mikhail Tsvet developed column chromatography, one of the traditional separation techniques, in the early 1900s [8]. Tsvet used petroleum ether as a mobile phase and calcium carbonate as a stationary phase to separate populated phytochromes [9]. Column chromatography was extensively used in the 1960s in the fields of biology, chemistry, medicine, and environmental protection. Monolithic wide-bore silica columns had been developed by Hironobu Morisaka et al. [10] in order to allow quick protein separation in accordance with molecular weights.; Quan Bai et al. [11] prepared a positively charged silica-based stationary phase modified with N-methylimidazolium IL for the separation of target proteins by adsorption of anisotropically charged proteins, and Hongjun Xia et al. [12] obtained chromatographic columns with higher separation efficiency than commercially available separation columns by controlling the structure and size of silica microspheres. However, the above-mentioned column’s adsorbent materials for protein separation have weak interactions with proteins, slow flow rates, and small specific surface areas. Therefore, the development of a strong cationic adsorbent material with high throughput is urgently needed.

Membrane chromatography has the advantages of mild conditions, high separation efficiency, simple operation, and small space occupation [13,14,15]. Ion chromatography belongs to a common separation technique in the membrane chromatography separation method, which is a separation technique using electrostatic gravitational force to achieve the separation purpose. The sulfonic acid group is a strongly interacting adsorption ligand that plays a significant role in the separation effect, and it has attracted considerable attention from researchers who want to combine them with porous materials for chromatography. The sulfonic acid–base chromatography material and the lysozyme can interact aggressively, which leads to a shorter duration of the lysozyme’s time of residence in the porous channels of membranes and enables rapid separation. Unfortunately, there is a poor link between the lysozyme and the weak cation exchange chromatography material, which prolongs the adsorption process. For example, Bo Feng et al. [16] utilized calcium lignosulfonate to separate scheelite and calcite; Bo Li et al. [17] prepared a nanoporous sulfonic acid covalent organic framework to separate lanthanides successfully. In addition, nanofibers are commonly exploited due to the advantages of tunable pore size, large specific surface area, easy functionalization, and simple fabrication [18,19,20,21]. Junying Yu et al. [22] prepared boron carbon nitride (BCN) nanofibers with a good adsorption effect on amino black 10B. Zhijiang Cai et al. [23] prepared polyindole nanofibers for the adsorption of Cu(II). Nanofibrous membranes have become a good candidate for chromatography materials with high separation performance.

In this paper, a novel sulfonic acid-based strong cationic protein adsorption membrane with a gradient structure was developed by the nanofiber wet-laying and grafting method. First, different extrusion speeds were used to modulate EVOH nanofibers with different diameters using the melt extrusion phase separation method; EVOH is a hydrophilic water-insoluble polymer also called poly(vinyl alcohol-co-ethylene) (PVA-co-PE), which possesses abundant hydroxyl groups beneficial to the surface modification [24]. EVOH nanofibrous membranes with both gradient or homogenous porous structures were prepared by a layer-by-layer wet-laying process of nanofibers with different diameters. Second, UV-assisted grafting was used to modify nanofiber layers with 2-acrylamido-2-methylpropanesulfonic acid as a modified monomer and benzophenone as a photoinitiator to fabricate a nanofibrous cation exchange chromatography membrane for lysozyme separation and purification. The static and dynamic adsorption capacities of the membranes with a gradient porous structure were higher than those of the membranes with a homogenous porous structure. The pore size, porosity, and specific surface area have been investigated to study their impact on adsorption performance. It was demonstrated that these factors worked together to produce these results. This creates new opportunities for the development of membrane materials for cationic protein adsorption separation.

## 2. Experiment

### 2.1. Materials and Reagents

Ethylene vinyl alcohol copolymer (EVOH), 44%, purchased from Sigma-Aldrich (Milwaukee, WI, USA); Cellulose acetate butyrate (CAB), 381-20, purchased from Acros Chemical (Pittsburg, PA, USA); acetone, industrial grade, purchased from Wuhan Xinmin Chemical Co., Ltd., Wuhan, China; deionized water, grade I, homemade in the laboratory; isopropanol, N,N-Dimethylformamide (DMF), anhydrous ethanol, sodium hydroxide, sodium chloride, sodium dihydrogen phosphate, disodium hydrogen phosphate, phosphoric acid, analytically pure, purchased from Sinopharm Chemical Reagent Corporation; Benzophenone (BP), 2-acrylamido-2-methylpropanesulfonic acid (AMPS), 99%, purchased from Bailing Wei Technology Co. Materials Co Ltd.; Lysozyme, purchased from Sigma Chemical Co (St. Louis, MI, USA). A commercial chromatography column (NW Rose Plus SP HP, 25 mL) was purchased from Suzhou NanoMicro Technology Co., Ltd., Suzhou, China.

### 2.2. Preparation of Nanofibrous Chromatographic Membranes with Homogenous Porous Structure

**Figure 1:**
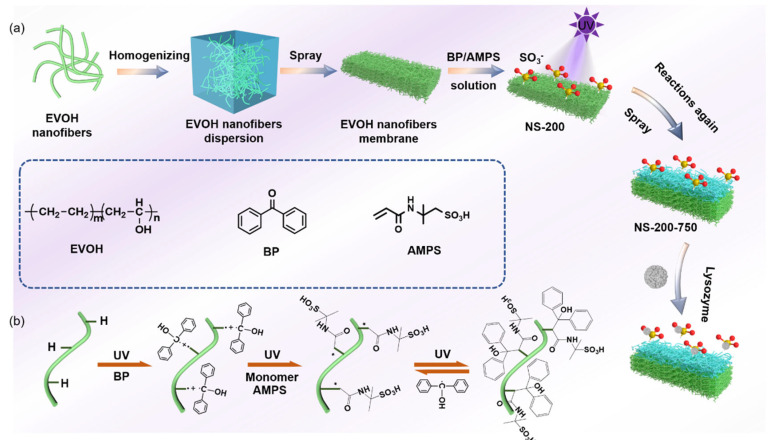
(**a**) Schematic preparation route of NS-X fiber membrane; (**b**) Reaction formula of nanofibers with BP/AMPS.

Nanofibers were prepared using the method reported in our previous work [24]^.^ The homogenous nanofibrous membranes without graft modification were fabricated via coating nanofiber suspension on a smooth surface, as shown in Figure 1a. Membranes of nanofibers with different diameters were named N-X (X is the fiber diameter). Later, a mixed solution of BP and AMPS with different concentrations was configured, and DMF was used as the solvent. Then, nitrogen was used to conduct the bubbling treatment to remove the oxygen in the solution, and the nanofibrous membrane of a certain size was cut to soak in the solution, completely wetted and taken out, and finally irradiated with UV light for a certain period of time. At the end of the reaction, the nanofibrous membrane was washed several times with ethanol and deionized water and dried in air to obtain homogenous nanofibrous membranes with sulfonate modifications (NS-X).

### 2.3. Preparation of Nanofibrous Chromatographic Membranes with a Gradient Porous Structure

In order to further improve the adsorption performance, a nanofibrous membrane with a gradient porous structure was proposed in this study. First, a nanofibrous membrane with fine nanofibers was prepared as a lower layer and further modified using the same method mentioned in Section 2.2. Then, the membrane was further coated by coarse nanofibers (as an upper layer) to obtain a nanofibrous membrane with a gradient pore size transmembrane. The upper layer was modified consequently to obtain the resultant nanofibrous chromatographic membranes (Figure 1a). They were named NS-X-Y(N) (X is the nanofiber diameter of the lower layer; Y is the nanofiber diameter of the upper layer, (N is the coating density of the upper layer of nanofibers, in g/m^2^). Nanofibers with average densities of 1 g/m^2^, 2 g/m^2^, 4 g/m^2,^ and average diameters of 762.6 nm were coated on nanofibers with average diameters of 264.5 nm, named NS-200-750(1), NS-200-750(2), NS-200-750(4). In this article, the NS-200-750 (2) defaults to the NS-200-750.

### 2.4. Lysozyme Adsorption Performance Test

#### 2.4.1. Determination of Static Lysozyme Adsorption Performance

Based on the negatively charged sulfonate ions on the surface of the fiber, the membrane will have electrostatic attraction with the positively charged amino groups on the protein. In this work, lysozyme with an isoelectric point of 10–11 was adopted to investigate the protein adsorption performance of the nanofibrous membrane. First, an aqueous buffer solution of 0.2 M NaH_2_PO_4_ and 0.2 M Na_2_HPO_4_ was prepared with a pH value of 5. A certain amount of lysozyme was weighed and added to the solution, which was stirred slowly until it was homogeneous. A nanofibrous chromatographic membrane of 50 mg was added to 15 mL of 2 mg/mL of lysozyme solution. The concentrations of lysozyme solutions before and after adsorption were measured using a UV spectrophotometer (UV, UV-27100, Shimadzu Instruments (Suzhou) Ltd., Suzhou, China).

The fiber membrane’s adsorption capacity was calculated by Equation (1).

(1) $$q^{*}=\frac{\left(C_{0}-C_{1}\right)*V}{m_{0}}$$

where V is the lysozyme volume, m_0_ is the fiber membrane mass, q^*^ is the fiber membrane’s lysozyme adsorption capacity (mg/g), C_0_ is the lysozyme beginning concentration, and C_1_ is the lysozyme final concentration.

#### 2.4.2. Determination of Dynamic Lysozyme Adsorption Performance

The homemade column was obtained by loading 8 sheets of the chromatographic membrane with a 50 mm diameter syringe filter under pressure. Lysozyme solution (2 g/L and pH 5) was injected continuously through the chromatographic column until the concentration of filtrate was the same as the concentration of feed solution.

#### 2.4.3. Evaluation of Recycling Effectiveness

The recycling protein adsorption–desorption performance of the chromatographic membrane and column was evaluated by conducting the above-mentioned test for five rounds. In order to remove the lysozyme that had adhered to the fiber membrane after each round, the adsorbed membrane was first rinsed with phosphate buffer containing 1 M NaCl. This process continued until the lysozyme absorption peak was no longer visible. After rinsing the fiber membrane with deionized water to remove the adsorbed NaCl solution, the next adsorption–desorption process was put aside for use in more adsorption tests.

### 2.5. Testing and Characterization

The chemical structure, composition, and surface electric potential of the membrane were probed using Fourier Transform Infrared Spectrometer (FTIR, Tensor 27, Bruker Optics, Ettlingen, Germany), X-ray photoelectric energy spectrometer (XPS, Thermo Scientific K-Alpha, Thermo Fisher, Waltham, MA, USA), and solid zeta potential (Zeta, SurPASS3, Anton Paar, Graz, Austria), respectively. The hydrophilicity of the samples was characterized using a water contact angle tester (WCA, DSA300S, KRUSS Scientific Instrument (Shanghai) Co, Ltd., Shanghai, China). The surface morphology of the membrane samples was analyzed by scanning electron microscopy (SEM, JEOL JSM-6510L, Tokyo, Japan). Prior to characterization, the samples were dried and sprayed with platinum for 240 s. The water permeability of the membrane was evaluated in a microfiltration membrane separation apparatus (XW-316, Faith&Hope Membrane Technology Co, Ltd., Suzhou, China). The pore size distribution and porosity of the membranes were measured by using a capillary flow porometer (Model CFP-1500A, PMI Inc., Los Angeles, CA, USA) and gas pycnometer (Micromeritics, AccuPyc II1340, McMuratic (Shanghai, China) Instrument Co., Ltd., Shanghai, China), respectively. Using a fully automated specific surface and porosity analyzer (BET, Mike ASAP2460, Shanghai-based McMurray Tic Instruments Co., Shanghai, China), the specific surface area of the fiber membrane was measured.

## 3. Results and Discussion

Figure 1b presents a detailed route for the preparation of NS membranes preparation. The grafting reaction is divided into two steps [25]. In the first step, benzophenone generates benzophenone radicals under UV light irradiation, which seize the hydrogen atoms on the nanofiber membrane to form a surface photoinitiator. In the second step, the initiator is then grafted and polymerized with the monomer (AMPS) under UV light conditions to the chromatographic membrane with a sulfonate surface. In addition, this reaction also produces an adverse reaction, where -OH on the nanofiber membrane is converted to -C=O (Figure S1). In order to determine the optimum grafting condition, we employed membranes with an average nanofiber pore size of 763.5 nm to improve the reaction time frame, BP concentration, and AMPS concentration. The results showed that the best adsorption performance was obtained when the membrane was modified under the grafting condition at twenty minutes of reaction time, 2.5% BP concentration, and 20% AMPS concentration (Figures S2–S4 in the Supporting Information), which was finally adopted to prepare membranes with other porous structures.

**Figure 2:**
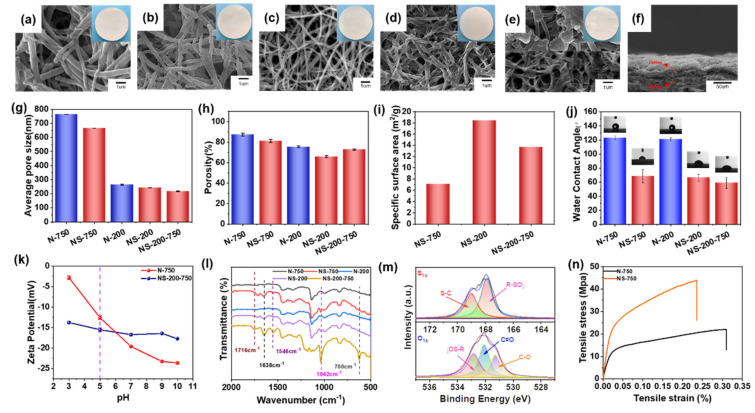
Surface morphology and physicochemical properties. Top-view SEM images of (**a**) N-750, (**b**) NS-750, (**c**) N-200, (**d**) NS-200, and (**e**) NS-200-750. The cross-sectional SEM image of (**f**) NS-200-750. (**g**) average pore size, (**h**) porosity, (**i**) specific surface area, (**j**) water contact angle of membranes, (**k**) zeta potential, (**l**) FTIR, (**m**) S_1s_ and O_1s_ XPS spectra of NS-200-750 and (**n**) the tensile strength of N-750 and NS-750.

To systematically study the pore structure on the lysozyme separation property of surface-sulfonated nanofibrous membranes, the surface morphology, porosity, pore size, and surface features were investigated, as shown in Figure 2. Compared with that of N-200 and N-750, nanofibers of NS-200 and NS-750 are slightly rougher and coarser, tightly adhered with each other (Figure 2a–d), respectively, suggesting the abundant grafting reaction, which also improves the mechanical property of nanofibrous membrane (Figure 2n). Due to the above-mentioned fiber morphology change, the pore size and porosity of NS-200 and NS-750 both decreased compared with their neat control (Figure 2g,h). For NS-750, the porosity decreased to 81.21%, and the average pore diameter decreased to 666.1 nm. For NS-200, the porosity and pore size were reduced to 65.91% and 243.2 nm from a high level separately.

The adsorption and desorption process for chromatography separation depends on the pore size transmembrane, which is homogenous for the above-mentioned NS-200 and NS-750 but was not the best choice in this study due to the difficulty of transferring the solution in the deep and small pores. To solve this issue, we think the better option would be the gradient porous structure, which possesses a larger pore size in the upper layer but a smaller pore size in the lower layer. As shown in Figure 2e, the upper surface and lower surface morphology of NS-200-750 are similar to that of NS-750 and NS-200, respectively. The cross-section view exhibits typical bi-nanofiber-layer morphology and the gradient pore structure (Figure 2f). Nanofibrous chromatographic membranes with different porous structures present an obvious distinction among the pore size, porosity, and even specific surface area (see the red column in Figure 2g–i). NS-200-750 shows the smallest transmembrane pore size compared with that of NS-750 and NS-200, which can be assigned to the smaller diameter of the fiber and larger thickness of the membrane via nanofiber stacking layer-by-layer. NS-200-750 with 750 nm nanofiber coverage density of 1, 2, and 4 g/m^2^ are found to be 236.5 nm, 217.1 nm, and 204.9 nm, respectively (Figure S5), indicating the higher fiber coverage density (larger thickness), the smaller pore size the membrane is. It should be noted that the static adsorption capacity of NS-200-750(2) is greater than that of NS-200-750(1) and NS-200-750(4) (Figure S6); thus, NS-200-750(2) has been selected as the membrane sample for further analysis in our work. For porosity, NS-200-750 possesses a moderate value compared with NS-750 and NS-200, which can be attributed to the more pore space occupied by thinner nanofibers and tends to be tightly compacted under the capillary force during the drying process [26] and further generates lower porosity. In addition, the specific surface area of NS-200-750 is also located at an intermediate level among the three samples derived from the positive relationship between fiber diameter and specific surface area. In addition, NS-200-750 also presented a slightly higher tensile strength but 24% higher tensile strain compared with the homogenous ones NS-200 and NS-750, indicating the superior mechanical property of the membrane with a gradient porous structure (Figure S7).

Sulfonated nanofibrous membranes possess much smaller water contact angles than their control, implying the improved hydrophilicity and the successful grafting of sulfonic acid groups on the surface of nanofibers, as shown in Figure 2j. Despite the nanofiber diameter difference, NS-200 and NS-750 present the same water contact angles, but NS-200-750 has better hydrophilicity than NS-200 and NS-750, manifesting that the gradient transmembrane pore structure is beneficial to the infiltration of water into the pores. The surface zeta potential of NS-200-750 is smaller than N-750, which can be attributed to hydroxyl and sulfonic acid groups on the membranes (Figure 2k). The more stable surface zeta potential of NS-200-750 varied with pH and can be assigned to the coexistence of -NH and -SO_3_H, on which the protonation occurs in turn at different pH [27]. The chemical groups on the nanofibrous membrane surface have been detected via FTIR and XPS investigation (Figure 2l,m and Figure S8). In the FTIR spectra, comparably, NS-200, NS-750 and NS-200-750 exhibit new peaks at 1716 cm^−1^ (caused by stretching vibration of -C=O), 1638 cm^−1^ and 1546 cm^−1^ (stretching vibration of -C=O-NH-), 1042 cm^−1^ (stretching vibration of sulfonic acid group [28]), 700 cm^−1^ (bending vibration of C-H on the benzene), which all demonstrate the successful grafting of AMPS on membranes. The element S can also be found in XPS spectra for NS-200-750. Peaks belonging to C-S and S=O of sulfonic acid groups are observed in the deconvoluted XPS spectra [28] (Figure 2m). 

**Figure 3:**
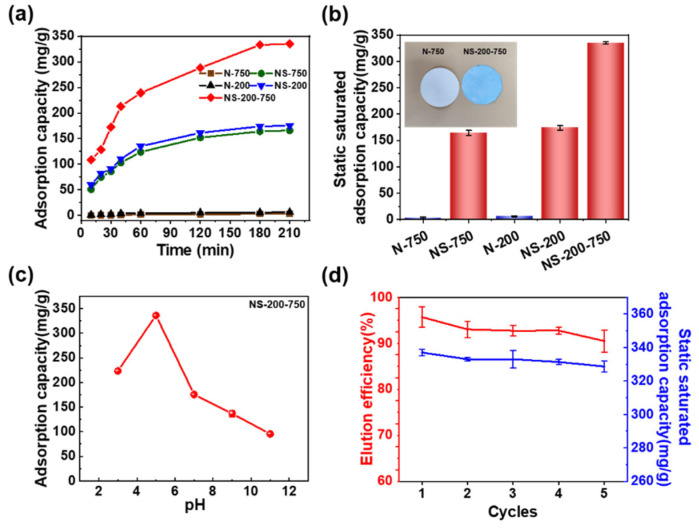
Lysozyme static adsorption capacities of nanofibrous membranes. (**a**) Static adsorption capacities with time and (**b**) static saturated adsorption capacities at pH = 5 with the optical photograph of N-750 and NS-750 adsorbing lysozyme and stained by Coomassie blue; (**c**) static saturated adsorption capacities with pH, and (**d**) repetitive adsorption capacities of NS-200-750 in 5 test cycles.

It is vital to investigate the adsorption behavior of nanofibrous membranes towards lysozyme. The adsorption of the nanofibrous membrane essentially reached saturation at about 180 min, as illustrated in Figure 3a. The nascent nanofibrous membrane shows near zero adsorption performance for lysozyme throughout the whole adsorption process, which is also indicated by the photograph of blue NS-200-750 and white N-750 (Figure 3b). NS-200-750 presents the best adsorption performance with a static adsorption saturation capacity of 335 mg/g compared with NS-750 (164.3 mg/g) and NS-200 (175.2 mg/g) (Figure 3a,b). Although NS-200 possesses a much smaller fiber diameter and much higher specific surface area than that of NS-750, the adsorption saturation capacity of the former is just slightly higher than the latter, which can be partially attributed to the smaller pore size of NS-200 than NS-750. This indicates that the pore structure for transferring the solution and lysozyme is a vital factor for a nanofibrous membrane to adsorb lysozyme in an aqueous solution, which is also why NS-200-750 with a gradient pore structure (Figure 2) presents the best adsorption property. We think that the gradient pore structure is beneficial to the transfer of lysozyme molecules from the solution to the surface of the active surface of nanofibers in the membrane, which will be deeply discussed.

The effect of the initial concentration of lysozyme on the adsorption capacity of NS-200-750 was further investigated. The supporting evidence (Figure S9) demonstrates how the lysozyme concentration affects the fiber membrane’s adsorption capacity, and when the lysozyme concentration reached 2 g/L, the adsorption capacity of the fiber membrane for lysozyme was 335 mg/g. The adsorption capacity of the fiber membrane was not significantly enhanced with a further increase in lysozyme concentration, and therefore, the subsequent experiments were carried out at 2 g/L lysozyme concentration. A pseudo-second-order model [29,30] was supported by the adsorption kinetics, while the lysozyme adsorption model on the fiber membrane followed the Langmuir isothermal adsorption model (Figure S10). The pH of the buffer solution influences how the lysozyme and nanofibrous membrane interact with each other, which in turn influences the adsorption performance of nanofibrous membranes in application. The membrane’s adsorption capacity towards lysozyme increased and then decreased as pH increased, with a maximum value of 335 mg/g at pH = 5 (Figure 3c). This is mostly because many hydrated hydrogen ions prevent the sulfonic acid groups in NS-200-750 from dissociating at low pH [31] and the decreased number of negative charges on the sulfonic acid groups. On the other hand, at high buffer pH, the number of positive charges carried on the surface of lysozyme decreased, which further weakens the strength of the electrostatic adsorption between lysozyme and nanofibers. The reusability of membranes is a crucial criterion in assessing their real applications. After five cycles of adsorption–desorption, the NS-200-750 still shows high adsorption capacity, and high desorption efficiency exceeds 90%, as shown in (Figure 3d) implying the stability of structure and adsorption property of the membrane.

**Figure 4:**
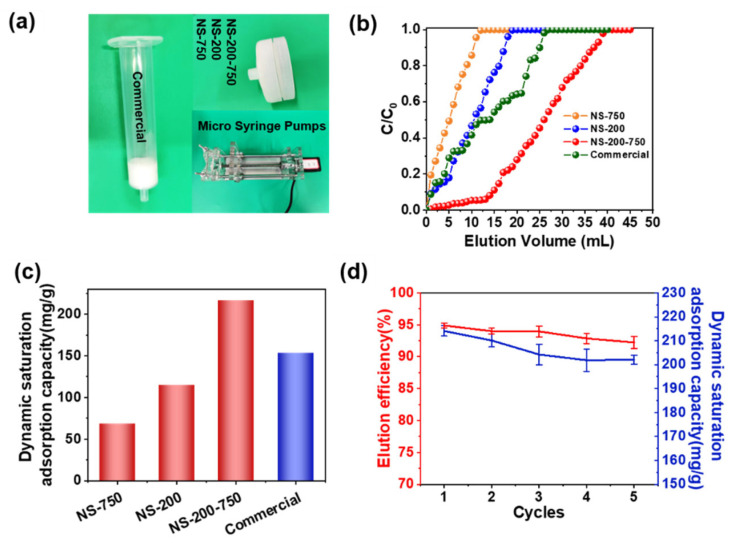
Dynamic lysozyme adsorption performance of chromatography column. (**a**) Optical photographs of NS-750, NS-200, NS-200-750 chromatography column, commercially available chromatography column, and syringe pump for testing dynamic adsorption, (**b**) dynamic adsorption breakthrough curves, and (**c**) dynamic adsorption capacity of chromatography columns; (**d**) repetitive adsorption–desorption capacities graph of NS-200-750 chromatography column in 5 dynamic test cycles.

A crucial measurement for assessing the practical application potential of protein-adsorbent materials is their dynamic adsorption capacity [32,33]. In this work, nanofiber membranes’ dynamic adsorption capabilities were determined by injecting lysozyme solution through a syringe filter with the assistance of a syringe pump, as shown in Figure 4a. Figure 4b shows that all chromatography columns presented typical lysozyme adsorption breakthrough curves at the flow rate of 1.5 mm min^−1^. When the concentration of the effluent lysozyme solution reached the initial value (*c*/*c_0_* = 1), the elution solution volumes of 11 mL, 18 mL, 26 mL, and 40 mL were obtained for the NS-750 column, NS-200 column, commercial column, and NS-200-750 column (Figure 4b), respectively. The dynamic saturation adsorption capacity of the NS-200-750 column was finally acquired as 216.3 mg/g higher than other columns (Figure 4c) and a stable adsorption performance even after five cycles of the adsorption–desorption process with a desorption efficiency of over 90% (Figure 4d).

**Figure 5:**
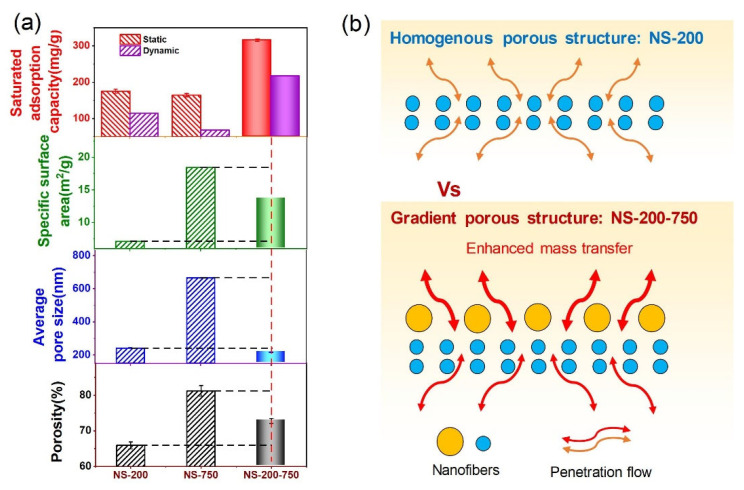
(**a**) Relationship between saturated adsorption capacity and structure parameters: porosity, average pore size, and specific surface area for NS-750, NS-200, and NS-200-750; (**b**) Schematic illustration for explaining the mass transfer behavior in diverse porous structures.

We summarized the relationships between adsorption capacities and porosity, specific surface area, and pore size (Figure 5a). It was found that none of them directly determines the adsorption capacity. Therefore, to explain the reason that NS-200-750 possessed a superior adsorption performance than other nanofibrous chromatographic membranes, we proposed a gradient porous structure mechanism. We think that the gradient porous structure is beneficial to the transfer of lysozyme molecules from the solution to the surface of the active surface of nanofibers in the membrane. The gradient porous structure of NS-200-750 holds the smaller inner pores made by nanofibers with a diameter of about 200 nm and the larger outer pores formed by nanofibers with a diameter of around 750 nm. This structure presents two merits for the capture and release of proteins. The first one is that the larger pores outside can enhance the convective transfer of solution, as indicated by the water flux test of NS-200-750 with a value of 4455 L h^−1^ m^−2^, which is 26.7% higher than NS-200 (Figure S11). The other one is that the inner pores of NS-200 can be easily blocked for further diffusion after the outer surface captures the protein molecule. However, for NS-200-750, nearly all active sites are open for diffusion without few or no obstacles. The improved convective and diffusion transfer caused by the gradient porous structure jointly enables a high protein capture and release rate (Figure 5b).

This develops as a consequence of the upper layer’s macroporous structure, which increases the lysozyme’s mass transfer rate (Figure 5b). This gradient structure is the outcome of the combined influence of porosity, average pore diameter, and specific surface area instead of each interacting separately. Whereas NS-200-750’s porosity, average pore diameter, and specific surface area fall between NS-200 and NS-750, its dynamic and static adsorption effects are superior (Figure 5a).

## 4. Conclusions

In summary, nanofibrous chromatographic membranes have been fabricated via a layer-by-layer wet-laying process of nanofibers and followed by UV-assisted grafting 2-acrylamido-2-methylpropanesulfonic acid initiated by benzophenone. As-prepared membranes with a gradient and homogenous porous structures were adopted to extract lysozyme protein from an aqueous solution. It was found that high specific surface area, small pore size, or high porosity does not directly generate the highest adsorption capacity, but the gradient porous structure is a determining factor. We think that the conclusion is novel and important for developing advanced next-generation chromatographic membranes with high adsorption performance. In our future work, the nanofibrous chromatographic membrane with more fiber layers and higher separation properties will be prepared and systemically studied.

## Data Availability

The data presented in this study are available on request from the corresponding author due to legal reason.

## Supplementary Materials

The following supporting information can be downloaded at: https://www.mdpi.com/article/10.3390/polym16081112/s1. Figure S1. Reaction formulae for side reactions. Figure S2. (a) SEM images of NS-750-20 min, (b) FTIR, (c) water contact angles, (d) average pore size, (e) water flux under 0.5 bar transmembrane pressure, (f) adsorption capacity for lysozyme of membranes with diverse AMPS grafting reaction time. Figure S3. (a) The SEM images of NS-750-2.5%BP, (b) FTIR, (c) water contact angles, (d) average pore size, (e) water flux under 0.5 bar transmembrane pressure, (f) adsorption capacity for lysozyme of membranes with diverse BP grafting concentration. Figure S4. (a) The SEM images of NS-750-20%AMPS, (b) FTIR, (c) water contact angles, (d) average pore size, (e) water flux under 0.5 bar transmembrane pressure, (f) adsorption capacity for lysozyme of membranes with diverse AMPS grafting concentration. Figure S5. Average pore size and porosity of NS-200-750 with 750 nm nanofiber coverage density of 1, 2, and 4 g/m2 corresponding to NS-200-750(1), NS-200-750(2), NS-200-750(4), respectively. Figure S6. Static adsorption capacity of NS-200-750(1), NS-200-750(2), NS-200-750(4). Figure S7. (a)The tensile strength and (b)the tensile strength per gram of NS-750, NS-200, and NS-200-750. Figure S8. XPS spectra of N-750 and NS-200-750. Table S1 Langmuir and Freundlich fitting parameters for the adsorption process of lysozyme by NS-200-750. Table S2 Pseudo-first-order and Pseudo-second-order model fitting parameters for the kinetic lysozyme adsorption process on NS-200-750. Figure S9. The adsorption capacity of NS-200-750 varied with lysozyme concentration. Figure S10. (a) Langmuir and (b) Freundlich isotherm plots of NS-200-750 for the adsorption of yeast lysozyme. (c) Pseudo-first and (d) pseudo-second-order kinetic plots of NS-200-750 for the adsorption of yeast lysozyme. Figure S11. Water flux of membranes under a trans-membrane pressure difference of 0.5 bar.
