# Supplementary material for: Ethylene Vinyl Alcohol Copolymer Nanofibrous Cation Exchange Chromatographic Membranes with a Gradient Porous Structure for Lysozyme Separation
Source: Polymers (Basel). 2024 Apr 16;16(8):1112. doi: 10.3390/polym16081112 (PMC11054456; doi:10.3390/polym16081112)
Supplement: Supplementary file 1 [file polymers-16-01112-s001.zip › polymers-2949067-supplementary.pdf]

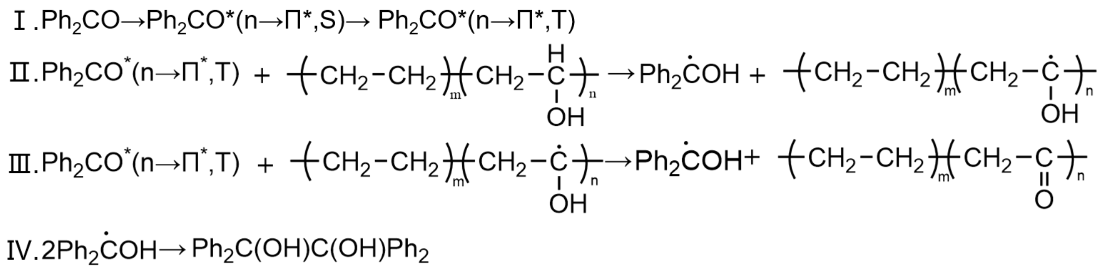

Figure S1. Reaction formulae for side reactions.

Impact of reaction time on NFM performance

UV graft-modified nanofibrous membranes were employed to optimize the reaction time, BP concentration and AMPS concentration, and the effect of reaction time on N-750 performance was firstly explored. EVOH nanofiber membranes with an average diameter of 762.6 nm, 10% BP and AMPS were firstly utilized as reaction conditions to explore the optimal reaction time. The grafted nanofibers had particulate matter adhering to the surface (Fig.S2a)，(Fig. S2b) shows the FTIR of N-750 as well as NS-750 with different reaction times, new FTIR absorption peaks appeared at 1638 cm^{-1} for the stretching vibrational absorption peak of -C=O-NH-; at 1042 cm^{-1} for the vibrational absorption peak of the sulfonic acid group[1]; and at 700 cm^{-1} for the bending vibrational absorption peak caused by C-H on the benzene, which proved the successful grafting of AMPS. Increasing hydrophilicity and decreasing pore size due to access of sulfonic acid groups (Fig. S2c,d),At the same time, the water flux was reduced at a transmembrane pressure difference of 0.5 bar(Fig. S2e). The Most importantly, the adsorption capacity of the fiber membrane for lysozyme changed with the increase of reaction time, as shown in (Fig. S2f). Among them, 71 mg/g for reaction 5 min; 95.3 mg/g for reaction 10 min; 103.3 mg/g for reaction 15 min; 121.3 mg/g for reaction 20 min; and 121.7 mg/g for reaction 25 min. When the reaction time was 20 min, stabilization was basically reached, so subsequent experiments were carried out at 20 min.

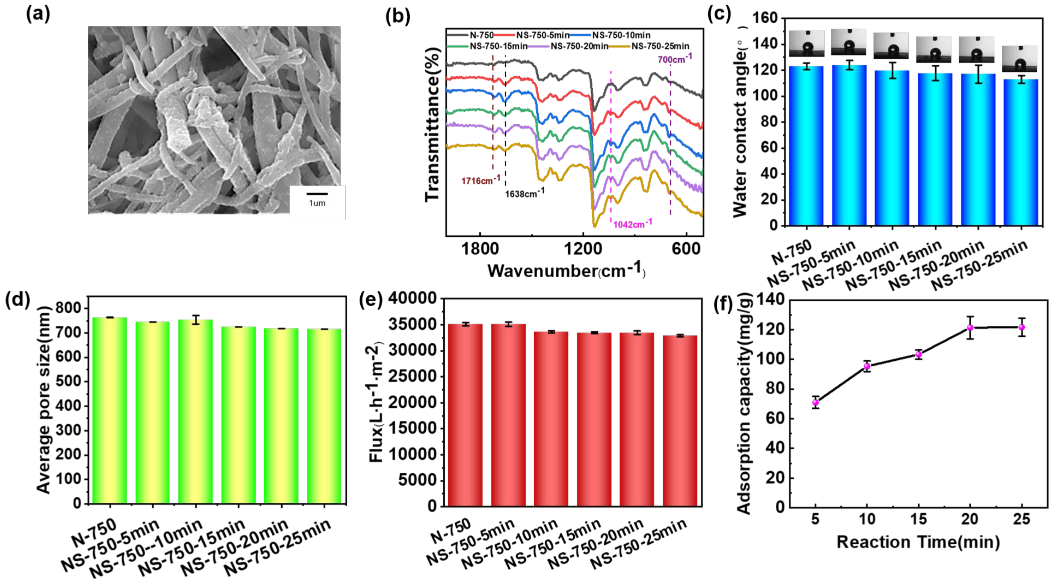

Figure S2. (a) SEM images of NS-750-20min, (b) FTIR, (c) water contact angles, (d) average pore size, (e) water flux under 0.5 bar transmembrane pressure, (f) adsorption capacity for lysozyme of membranes with diverse AMPS grafting reaction time.

Impact of BP concentration on NFM performance

Secondly, the effect of BP concentration on the adsorption performance as well as other properties was further explored in this paper. The grafted nanofibers had particulate matter adhering to the surface (Fig. S3a),(Fig. S3b) shows the FTIR of N-750 as well as NS-750 with different BP concentration, new FTIR absorption peaks appeared at 1638 cm^{-1} for the stretching vibrational absorption peak of -C=O-NH-; at 1042 cm^{-1} for the vibrational absorption peak of the sulfonic acid group [1]; and at 700 cm^{-1} for the bending vibrational absorption peak caused by C-H on the benzene, which proved the successful grafting of AMPS. With the increase of BP concentration, the water contact angle, average pore size, and water flux showed a trend of decreasing and then increasing (Fig. S3c,d,e). When the concentration of BP was 2.5%, the hydrophilicity of the fiber membrane was the best, and the adsorption capacity of lysozyme was the strongest, up to 140 mg/g (Fig.S3f). The above results indicate that lower photoinitiator is not sufficient to produce enough free radicals to initiate the reaction, whereas too much initiator produces too many free radicals, which can lead to further occurrence of free radical coupling reaction and free radical chain transfer reaction or free radical as impurity inhibition reaction. Therefore, the free radical reaction requires a suitable amount of initiator, and when the photoinitiator reaches a certain amount, increasing the amount of initiator again does not greatly increase the conversion and grafting of AMPS.

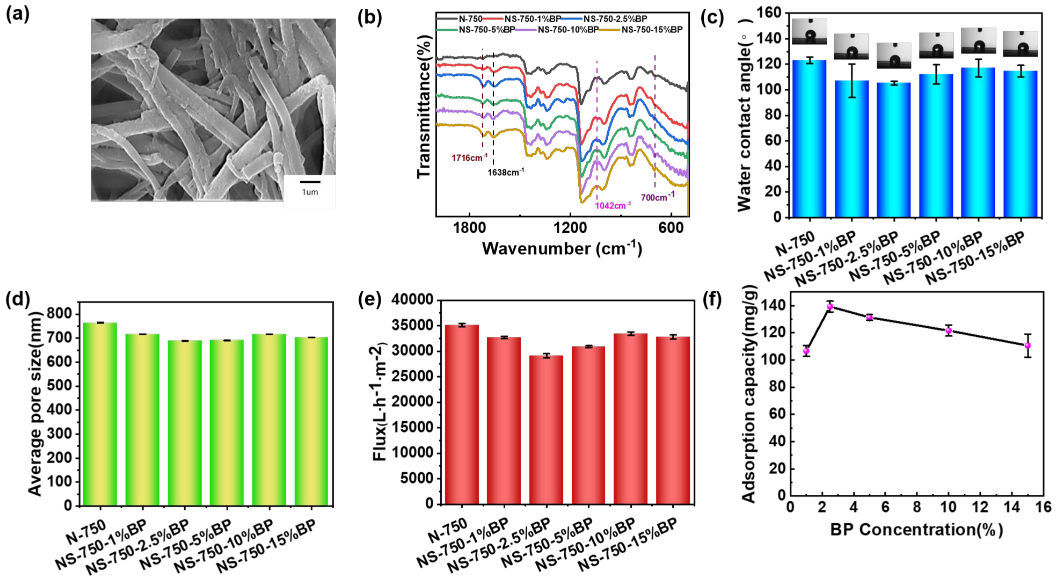

Figure S3. (a) The SEM images of NS-750-2.5%BP, (b) FTIR, (c) water contact angles, (d) average pore size, (e) water flux under 0.5 bar transmembrane pressure, (f) adsorption capacity for lysozyme of membranes with diverse BP grafting concentration.

Impact of AMPS concentration on NFM performance

Finally, the effect of AMPS concentration on the adsorption performance as well as other properties was further explored in this paper. The grafted nanofibers had particulate matter adhering to the surface (Fig.S4a)， (Fig. S4b) shows the FTIR of N-750 as well as NS-750 with different BP concentration, new FTIR absorption peaks appeared at 1638 cm^{-1} for the stretching vibrational absorption peak of -C=O-NH-; at 1042 cm^{-1} for the vibrational absorption peak of the sulfonic acid group [1]; and at 700 cm^{-1} for the bending vibrational absorption peak caused by C-H on the benzene, which proved the successful grafting of AMPS. As the concentration of AMPS increased, the grafting rate became higher, the hydrophilicity became stronger, the average pore size became smaller, and the water flux decreased (Fig. S4c,d,e). Stabilization was reached when the AMPS concentration was 20%, at which time the static saturated adsorption capacity for lysozyme could reach 164 mg/g (Fig. S4f). The above results indicated that the free radical conversion rate of AMPS and the efficiency of light-induced generation of free radicals were approximately the same when the reaction concentration of AMPS was 20%, and the maximum reaction efficiency was reached.

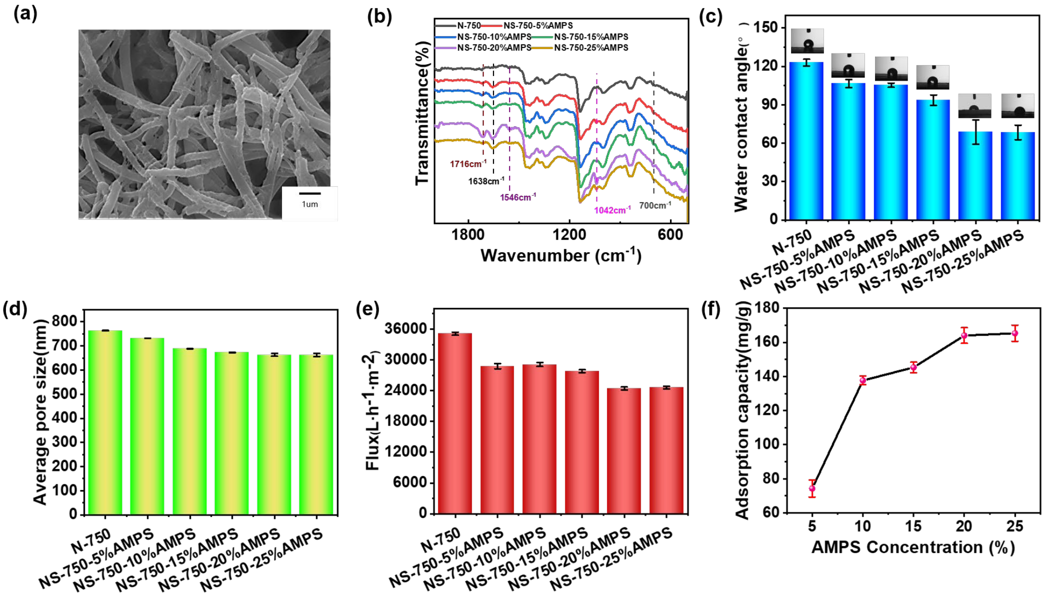

Figure S4. (a) The SEM images of NS-750-20%AMPS, (b) FTIR, (c) water contact angles, (d) average pore size, (e) water flux under 0.5 bar transmembrane pressure, (f) adsorption capacity for lysozyme of membranes with diverse AMPS grafting concentration.

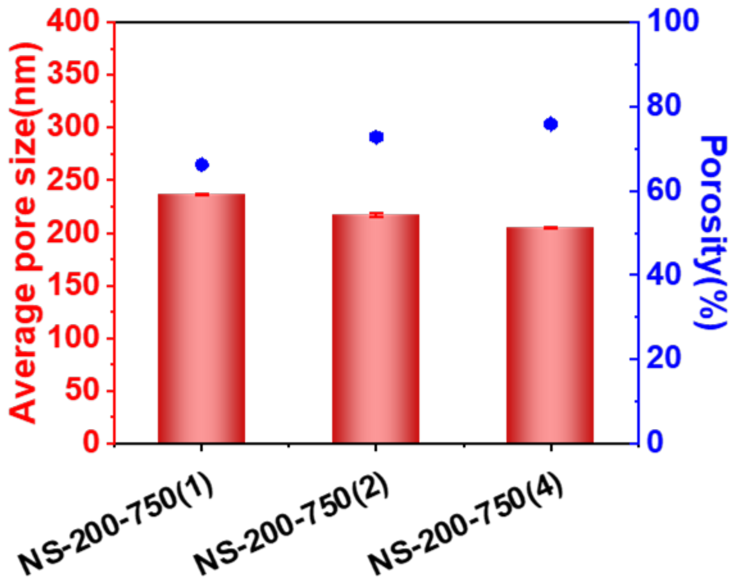

Figure S5. Average pore size and porosity of NS-200-750 with 750 nm nanofiber coverage density of 1, 2, and 4 g/m^2 corresponding to NS-200-750(1), NS-200-750(2), NS-200-750(4), respectively.

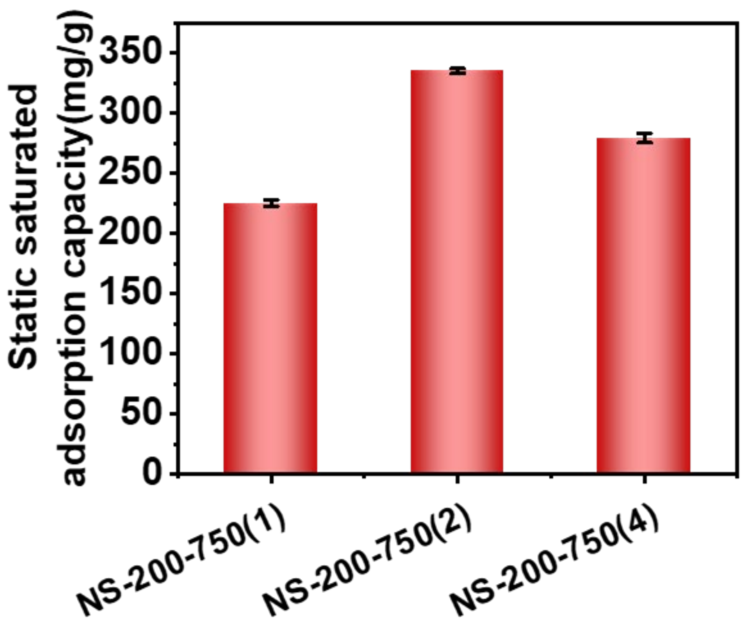

Figure S6. Static adsorption capacity of NS-200-750(1), NS-200-750(2), NS-200-750(4).

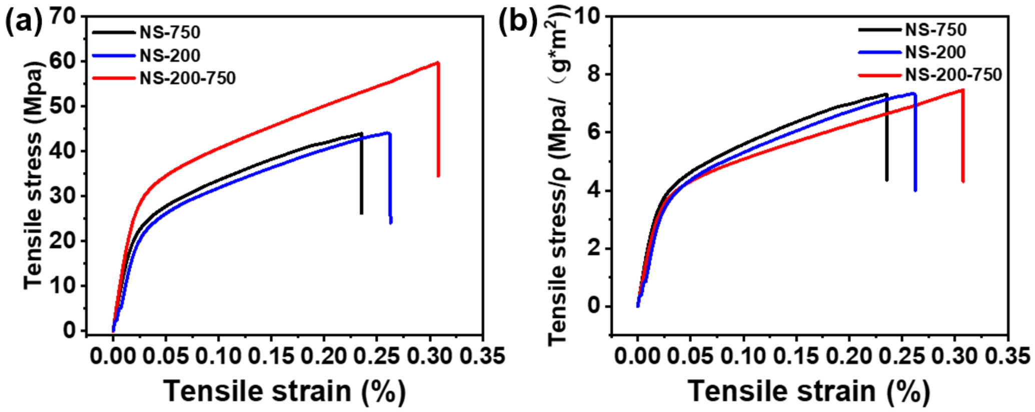

Figure S7. (a)The tensile strength and (b)the tensile strength per gram of NS-750, NS-200and NS-200-750.

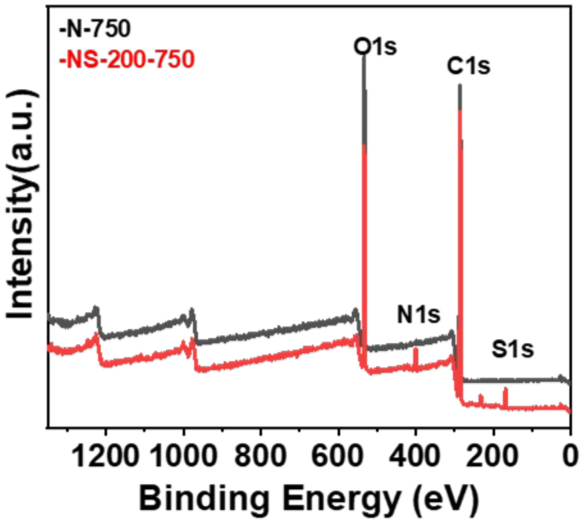

Figure S8. XPS spectra of N-750 and NS-200-750.

Isothermal adsorption process and adsorption kinetic process studies

The Langmuir, Freundlich isotherm, pseudo-first-order and pseudo-second-order model equations are as following[2][3]:

Langmuir isotherm:

$$\frac{1}{q_e}=\frac{1}{q_m}+\frac{1}{K_L q_m C_e}$$

where q_e is the adsorption capability of adsorbed lysozyme (mg/g) by NS-200-750, q_m is the maximum adsorption capacity for monolayer coverage (mg/g), K_L is Langmuir adsorption constant related to heat of adsorption, C_e is initial concentration of pollutants in solution.

Freundlich isotherm:

$$lnq_e = {}^{1}/_{n}\, lnC_e + lnK_F$$

where q_e is the adsorption capability of adsorbed lysozyme (mg/g) by NS-200-750, C_e is initial concentration of lysozyme in solution, K_F and n are the Freundlich constant and adsorption intensity, respectively.

Pseudo-first-order model is shown in following equation:

$$\ln(q_e - q_t) = lnq_e - k_1 t$$

where q_e (mg/g) denotes the calculate adsorption capacity of NS-200-750 and q_t (mg/g) denotes the amount of lysozyme adsorbed by NS-200-750 at a given time t, while k_1 (min^{-1}) is the pseudo-first-order rate constant for adsorption of lysozyme.

Pseudo-second-order model was shown in following equation:

$$\frac{t}{q_t}=\frac{t}{q_e}+\frac{1}{k_2 * q_e^2}$$

where q_e (mg/g) denotes the calculate adsorption capacity of NS-200-750 and q_t (mg/g) denotes the amount of lysozyme adsorbed by NS-200-750 at a given time t, while k_2 (min^{-1}) is the pseudo-second-order rate constant for adsorption of lysozyme.

As shown in (Fig. S9), the adsorption capacity of the fiber membrane increased with the increase of lysozyme concentration. In addition, the present study also explored the law of adsorption capacity of fiber membrane on lysozyme with time, as can be seen in (Fig. 3a), with the extension of time, the fiber membrane adsorbed rapidly in the first 180 min, and reached the adsorption equilibrium at 210 min. In order to investigate the adsorption mechanism of lysozyme on fiber membrane, we introduced Langmuir and Freundlich isothermal adsorption model, pseudo-first-order model and pseudo-second-order model for comprehensive analysis [3]. The fitted curves and related fitting parameters are shown in (Fig. S10a, b, c, d) and Table S1. The results showed that the capture capacity of the fiber membrane for lysozyme was more in line with the Langmuir isothermal adsorption model, and more importantly, the adsorption capacity of the fiber membrane for lysozyme calculated by the Langmuir isothermal adsorption model was 473.93 mg/g, which indicated that the further optimization of the physicochemical structure of the fiber membrane was expected to improve the adsorption performance of lysozyme. As can be seen from Table S2, relative to the quasi-primary kinetic equation (R^2=0.979), the adsorption process of lysozyme by the fiber membrane is more consistent with the quasi-secondary kinetic equation (R^2=0.995). Moreover, the adsorption capacity of lysozyme calculated by the quasi-secondary kinetic equation of 359.71 mg/g was more similar to that of 335 mg/g. The resulting NS-200-750 fast and easily tunable weakly physical adsorption makes it a promising candidate for the preparation of highly efficient chromatographic columns for various biological separations and purifications.

Table S1 Langmuir and Freundlich fitting parameters for the adsorption process of lysozyme by NS-200-750

Langmuir model			Freundlich model		
q_{max}(mg/g)	Ka(mg mL^{-1})	R^2	K_F	n	R^2
473.93	0.879	0.995	212.15	2	0.971

Table S2 Pseudo-first-order and Pseudo-second-order model fitting parameters for the kinetic lysozyme adsorption process on NS-200-750

Pseudo-first-order model			Pseudo-second-order model		
q_e (mg g^{-1})	k_1 (min^{-1})	R^2	q_e (mg g^{-1})	k_2 (min^{-1})	R^2
234.05	0.01257	0.979	359.71	0.000094	0.995

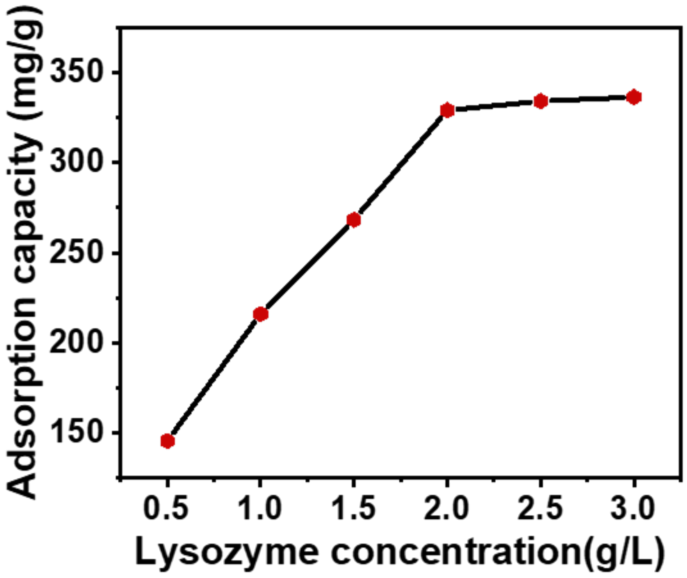

Figure S9. Adsorption capacity of NS-200-750 varied with lysozyme concentration

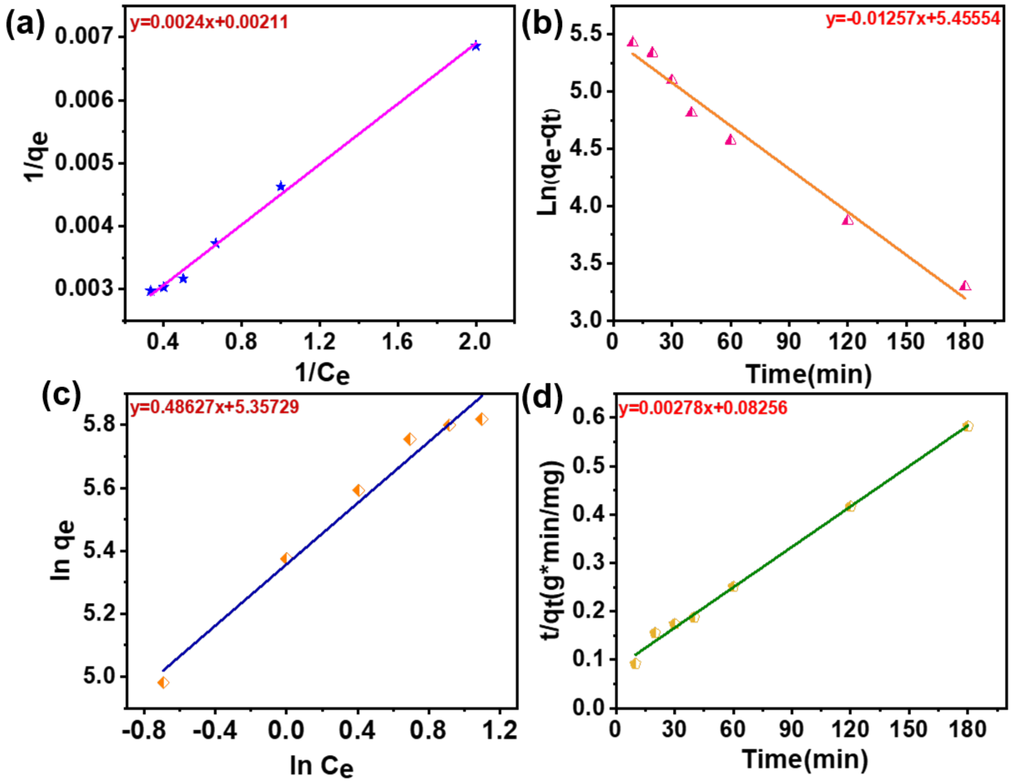

Figure S10. (a) Langmuir and (b) Freundlich isotherm plots of NS-200-750 for the adsorption of yeast lysozyme. (c) Pseudo-first and (d) pseudo-second order kinetic plots of NS-200-750 for the adsorption of yeast lysozyme.

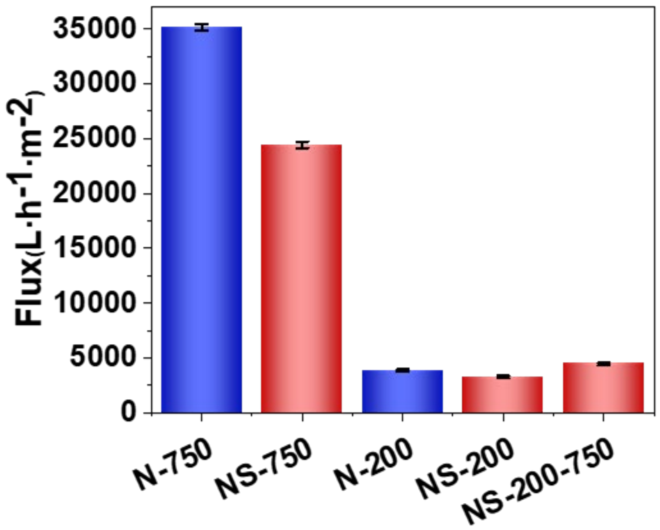

Figure S11. Water flux of membranes under trans-membrane pressure difference of 0.5 bar .

References

[1] Tran T H, Okabe H, Hidaka Y, et al. Removal of metal ions from aqueous solutions using carboxymethyl cellulose/sodium styrene sulfonate gels prepared by radiation grafting[J]. Carbohydrate polymers, 2017, 157: 335-343.

[2] Aguado-Deblas L, Estevez R, Russo M, et al. Microwave-assisted glycerol etherification over sulfonic acid catalysts[J]. Materials, 2020, 13(7): 1584.

[3] Shamsinar N, Saufi S M. Adsorptive cation exchanger mixed matrix membrane chromatography for the isolation of lysozyme from chicken egg white[J]. Arabian Journal for Science and Engineering, 2016, 41: 2479-2485.
